# Barriers to latent tuberculosis screening and treatment among people living with HIV in Brazil

**DOI:** 10.1590/0034-7167-2025-0416

**Published:** 2026-08-21

**Authors:** Eloisa Ganazza Mattera, Lucas Vinícius de Lima, Nelly Lopes de Moraes Gil, Aline Aparecida Monroe, Gabriel Pavinati, Gabriela Tavares Magnabosco

**Affiliations:** I Universidade Estadual de Maringá. Maringá, Paraná, Brazil; II Prefeitura do Município de Maringá, Secretaria Municipal de Saúde. Maringá, Paraná, Brazil; III Universidade de São Paulo. Ribeirão Preto, São Paulo, Brazil

**Keywords:** Latent Tuberculosis, HIV, Health Services, Preventive Therapy, Qualitative Research., Tuberculosis Latente, VIH, Servicios de Salud, Terapéutica Preventiva, Investigación Cualitativa.

## Abstract

**Objectives:**

to understand, from the perspective of professionals in specialized services, the aspects that influence latent *Mycobacterium tuberculosis* infection screening and treatment among people living with HIV.

**Methods:**

a qualitative study was conducted with 25 professionals prescribing preventive treatment from Manaus, Recife, Rio de Janeiro, Campo Grande, and Porto Alegre. Data were collected between 2024 and 2025 using an electronic questionnaire and analyzed using Bardin’s content analysis.

**Results:**

four categories were identified: Professional qualification and commitment (autonomy and resistance to prescription); Structure and resources for care (exams, supplies, and infrastructure); Treatment adherence and continuity of care (low adherence and clinical follow-up); and Health management and policies (shortage of professionals, turnover, and centralization).

**Final Considerations:**

challenges persist in implementing preventive tuberculosis treatment among people living with HIV in Brazil, including professional resistance to prescribing, training gaps, regional inequalities in access, and social vulnerabilities.

## RESUMEN

**Objetivos:**

comprender, desde la perspectiva de profesionales de servicios especializados, los aspectos que influyen en el cribado y el tratamiento de la infección latente por *Mycobacterium tuberculosis* en personas con VIH.

**Métodos:**

se realizó un estudio cualitativo con 25 profesionales que prescriben tratamiento preventivo en Manaus, Recife, Río de Janeiro, Campo Grande y Porto Alegre. Los datos se recopilaron entre 2024 y 2025 mediante un cuestionario electrónico y se analizaron mediante el análisis de contenido de Bardin.

**Resultados:**

se identificaron cuatro categorías: Calificación y compromiso profesional (autonomía y resistencia a la prescripción); Estructura y recursos para la atención (exámenes, insumos e infraestructura); Adherencia al tratamiento y continuidad de la atención (baja adherencia y seguimiento clínico); y Gestión y políticas de salud (escasez de profesionales, rotación y centralización).

**Consideraciones Finales:**

persisten desafíos en la implementación del tratamiento preventivo de la tuberculosis entre personas que viven con VIH en Brasil, incluyendo resistencia profesional a la prescripción, brechas de capacitación, desigualdades regionales en el acceso y vulnerabilidades sociales.

## INTRODUCTION

Tuberculosis (TB) is one of the major public health challenges in the world. Estimates from the World Health Organization indicate that approximately 25% of the global population is infected with Koch’s bacillus, but without active disease^(1)^. Latent *Mycobacterium tuberculosis* infection (LTBI), or TB-infection, is characterized by the host’s persistent immune response to the bacillus. Historically, LTBI has received less attention in TB control programs, hindering progress in reducing the chain of transmission^(1-3)^.

The literature suggests that people with LTBI act as a “reservoir of bacilli” with the potential for reactivation in situations of immunosuppression^(4,5)^. This risk is even more critical in people living with HIV, due to the immunodeficiency caused by the virus, which weakens the host’s immune response to the TB bacillus. In these individuals, TB not only represents the leading cause of death but also has a significant impact on public health, as it increases the potential for the disease to spread within communities^(5,6)^.

Preventive TB treatment (PTT) plays a central role, as it has the potential to reduce the risk of progression from LTBI to the active form of TB^(5)^. A scoping review indicated a reduction of between 27% and 95% in the incidence of TB-disease among people who received PTT compared to the placebo group, and the effectiveness depended on treatment duration and adherence rates^(7)^. This reiterates the importance of including PTT as a crucial strategy in addressing the global burden of TB^(7)^.

Despite the above, structural and operational obstacles related to healthcare services, such as distance to the service, lack of trained professionals, delays in care, diagnostic inaccuracy, low adherence to PTT, insufficient PTT coverage and insufficient financial resources, are some of the challenges pointed out in the literature worldwide^(8-11)^ that hinder the timely screening of LTBI and, consequently, PTT implementation, especially in more vulnerable populations, such as people living with HIV.

In Brazil, despite regulatory advances in expanding the range of professionals authorized to prescribe PTT-including nurses and pharmacists-, resistance to its implementation persists. This resistance stems not only from technical barriers but is also related to formative, organizational, and cultural issues that limit multidisciplinary practice and the effectiveness of health policies and strategies. Thus, it becomes essential to understand how current recommendations manifest themselves in the daily routine of healthcare services.

Therefore, understanding work processes in different Brazilian scenarios allows for the identification of critical points in the daily operations of services, as well as the planning of strategies specific to each reality. In this context, considering that the qualification of professionals is key to achieving the global goals of TB elimination, this research is justified by the need to support national actions aimed at expanding the coverage of TB vaccination screening among one of the priority populations in the TB elimination goals.

## OBJECTIVES

To understand, from the perspective of professionals in specialized services, the aspects that influence LTBI screening and treatment among people living with HIV.

## METHODS

### Ethical considerations

The research prioritized full compliance with the guidelines of Resolution 466 of December 12, 2012 and Resolution 674 of May 6, 2022, both from the Brazilian National Health Council. To this end, approval was obtained from the *Universidade Estadual de Maringá* Research Ethics Committee, in accordance with Opinion No. 6,899,096. Other authorizations were also obtained from the municipalities that required administrative and/or ethical procedures. All participants agreed to the Informed Consent Form for participation in the study.

### Study design and context

This is an exploratory study, using a qualitative approach, and reported in accordance with the COnsolidated criteria for REporting Qualitative research checklist^(12)^. For the operationalization of the study, Specialized Care Services (SCSs) located in five Brazilian capitals were listed, namely Manaus (Amazonas, in the North region), Recife (Pernambuco, in the Northeast region), Rio de Janeiro (Rio de Janeiro, in the Southeast region), Campo Grande (Mato Grosso do Sul, in the Midwest region) and Porto Alegre (Rio Grande do Sul, in the South region).

In Brazil, the treatment of people living with HIV is organized in a shared manner between primary health care and specialized care. However, there is still a noticeable centralization in SCSs, especially in cases of co-infection or multiple comorbidities. The SCS is an outpatient unit dedicated to the management of HIV and other sexually transmitted infections. Through multidisciplinary action, these services guarantee the prevention and treatment of affected individuals through specialized, comprehensive, and humanized care.

### Study period and participants

Data collection took place between November 2024 and February 2025. The study considered healthcare professionals from SCSs with a minimum of six months of experience in the service, who had provided at least one consultation to a person living with HIV and possessed training compatible with prescribing therapy (i.e., medicine, nursing, and pharmacy). It is noteworthy that this criterion aligns with the Ministry of Health’s current policy, which recognizes the possibility of physicians, nurses, and pharmacists advising on and prescribing PTT^(13,14)^.

### Data collection protocol

The instrument used to guide data collection was developed and adapted by researchers from the HIV/AIDS and Tuberculosis Surveillance Research Group, *Universidade Estadual de Maringá*, who were responsible for developing the study. It was designed to allow for participant characterization, who answered seven open-ended questions with the following central question: “How has the work process been regarding LTBI screening and PTT referral for people living with HIV in your SCS?”.

To recruit participants, more than 250 invitations were sent, considering messages sent directly to SCS professionals’ institutional contacts, obtained through the Ministry of Health’s website, in addition to the support of state coordinators of TB and HIV programs and municipal health secretariats for disseminating the research among SCS professionals. The self-administered instrument was made available virtually via Google Forms^®^, and, in the end, theoretical saturation was reached with 25 participants.

It is important to point out that responses to the questionnaire containing serious spelling or typing errors that compromised the partial or total interpretation of their content by the researchers were excluded from the study. In total, two responses to specific questions were disregarded for this reason. Finally, it is clarified that the text segments were identified according to the informant, represented by the letter P (participant), followed by an Arabic numeral (e.g., P1, P2 *etc*.), in order to preserve anonymity.

### Data processing and analysis

The text material obtained was analyzed using Bardin’s content analysis technique^(15)^. This analysis was anchored in three successive stages: pre-analysis, in which the materials were organized through a preliminary reading and subsequent hypothesis formulation; material exploration, identifying the units of recording (words) and context, through processes of decomposition, classification, grouping, and enumeration; and processing and interpretation of results, the moment when the final categorization was carried out.

## RESULTS

Of the 25 participants, the majority were female (n=23), had a nursing background (n=15), and were from Rio de Janeiro (n=12). Their ages ranged from 27 to 70 years (Chart 1). From the discourses, nine core meanings emerged-composed of constitutive elements (the professionals’ discourses)-, which culminated in four categories: Professional qualification and commitment (Chart 2); Structure and resources for care (Chart 3); Treatment adherence and continuity of care (Chart 4); and Health management and policies (Chart 5).

**Chart 1:** Research participant sociodemographic and occupational characteristic description, Brazil, 2025

ID	Sex	Age (in years)	Profession	Month and year of commencement of service	Capital
P1	Male	49	Nurse	10/2023	Manaus
P2	Male	44	Nurse	11/2005	
P3	Female	53	Nurse	02/2015	
P4	Female	44	Nurse	11/2015	Campo Grande
P5	Female	36	Pharmaceutical	11/2017	
P6	Female	36	Physician	07/2017	Porto Alegre
P7	Female	47	Nurse	10/2010	
P8	Female	64	Physician	05/2004	
P9	Male	38	Pharmaceutical	06/2024	Recife
P10	Female	27	Nurse	03/2022	
P11	Female	37	Nurse	07/2022	
P12	Female	27	Nurse	09/2023	
P13	Female	42	Nurse	01/2015	
P14	Female	64	Physician	11/1991	Rio de Janeiro
P15	Female	58	Nurse	10/2022	
P16	Female	27	Nurse	01/2024	
P17	Female	62	Nurse	09/2022	
P18	Female	48	Nurse	08/2013	
P19	Female	34	Physician	03/2019	
P20	Female	43	Nurse	02/2018	
P21	Female	28	Pharmacy	03/2024	
P22	Female	39	Nurse	07/2020	
P23	Female	63	Physician	02/2022	
P24	Female	40	Physician	02/2023	
P25	Female	70	Physician	01/2015	

**Chart 2:** Presentation of the core meanings and constituent elements of the “Professional qualification and commitment” category, Brazil, 2025

Constituent elements	Core meanings	Category
*A large part of the team is mobilized to pay attention to and monitor the patients.* (P9)	Professional commitment in providing healthcare is a strength in tuberculosis infection diagnosis and treatment	Professional qualifications and commitment
*I don’t have many problems with prescribing preventive treatment for tuberculosis; it is very well accepted and followed by professionals.* (P1)		
*There is great interest and involvement from the nursing team in latent infection control and management.* (P3)		
*Medication is offered to all patients who meet the indications, according to the protocol, because we screen all patients together with Primary Care and other healthcare services.* (P20)		
*Nurses provide care, case identification* [...], *treatment, monitoring, and active search for patients who miss appointments; this contributes to the service.* (P3)	Professional autonomy focused on tuberculosis infection screening and treatment by other professionals expands access to care	
*The autonomy of prescribing preventive treatment by professionals other than physicians, especially nurses, is positive.* (P7)		
*In our service, prescribing preventive treatment is a medical procedure, and not all colleagues follow the recommendations due to justifications associated with polypharmacy.* (P9)	Professional resistance to prescribing preventive tuberculosis treatment hinders compliance with current recommendations	
*Medical conduct regarding the prescription of preventive tuberculosis treatment is a challenge.* (P12)		
*Screening is not done as it should be* [...]; *I observe a “difficulty” by medical professionals in requesting screening for latent tuberculosis.* (P18)		

**Chart 3:** Presentation of the core meanings and constituent elements of the “Structure and resources for care” category, Brazil, 2025

Constituent elements	Core meanings	Category
*We have systematized monitoring methods in spreadsheets that help in the active search for those who interrupt treatment.* (P10)	(Un)availability of physical resources and care technologies is an indispensable part of providing quality care	Structure and resources for care
*The strong point is the monitoring of CD4 as an indicator for starting treatment.* (P1)		
*The incorporation of diagnostic tests* [for latent tuberculosis infection] *for people living with HIV expands access.* (P5)		
*We have constant availability of the necessary medications in our service* [...], *which is not a reality everywhere.* (P6)		
*I have quick access to imaging exams, such as X-rays and computed tomography, and laboratory exams, such as rapid molecular tests for tuberculosis, in addition to a trained health team.* (P8)		
*Having access to LF-LAM* [lateral flow test for detection of lipoarabinomannan in urine] *is a great advantage in the routine, but we still have difficulty with X-rays, as we need to keep referring to the regulatory system.* (P12)	(Un)availability of physical resources and care technologies is an indispensable part of providing quality care	Structure and resources for care
*We were detached from our unit and left in a house without conditions for the user to be fully attended to* [...]. *Our physician’s contract expired, and we are currently waiting for her to be rehired to continue treatments.* (P15)		
*We have a laboratory in epidemiology* [...], *we have several professionals who are multipliers of the tuberculin test. Test results are almost immediate. The multidisciplinary team integrates the specialized service and takes the necessary actions.* (P14)		
*The quantity of tablets* [...], *we still do not have the 3HP regimen available for all patients with the indication.* (P7)	Problems related to the availability of supplies and physical infrastructure hinder tuberculosis infection treatment	
*Unfortunately, we have inadequate physical space for the needs.* (P14)		
*Sometimes we do not have availability of tests such as the tuberculin test and IGRA* [interferon gamma release assay] *at the initial moment of care.* (P22)		
*Our physical space is restricted; this limits assistance.* (P20)		

**Chart 4:** Presentation of the core meanings and constituent elements of the “Treatment adherence and continuity of care” category, Brazil, 2025

Constituent elements	Core meanings	Category
*The challenge in screening is ensuring that patients have the requested chest x-ray.* (P2)	Low adherence to therapy among people living with HIV undermines the effectiveness of preventive treatment, compounding social vulnerabilities	Treatment adherence and continuity of care
*We have considerable difficulties, especially with those patients who are homeless.* (P3)		
*Patient adherence to treatment: this is a problem we have a lot of here.* (P8)		
[...] *there are cases of patients with difficult adherence.* (P10)		
*Despite the qualified team and availability of supplies, we have several patients who do not adhere to preventive treatment.* (P25)		
*The main challenge* [...] *is the observation of a patient after the start of therapy, especially those patients* [...] *with CD4 counts less than 350 cells/cubic millimeters.* (P6)	Challenges related to actively seeking out and counseling individuals using preventive treatment and their contacts	
*We usually conduct active searches only via cell phone, and this is not very effective* [...]; *phone numbers change a lot.* (P1)		
*Accessing and advising contacts is challenging, a difficulty.* (P24)		

**Chart 5:** Presentation of the core meanings and constituent elements of the “Health management and policies” category, Brazil, 2025

Constituent elements	Core meanings	Category
*Lack of human resources and qualified professionals.* (P3)	Insufficient numbers of professionals, with inadequate training and low qualifications, hinder the implementation of preventive treatment	Health management and policies
*Waiting time for an appointment can be long.* (P9)		
*Rotation of contracted professionals and periodic changes of professionals in the network.* (P8)		
*Newly graduated professionals have little knowledge about the subject, which impairs care.* (P8)		
*For me, I see a lack of knowledge when deciding on the indication for preventive treatment.* (P11)		
*We have a small team for the demand.* (P14)		
*We still need to improve a lot in the training of professionals.* (P16)		
*The fact that the provision of tuberculosis-infection care is centralized in points of the network is quite fragile.* (P7)	Political and organizational challenges hinder the provision of services and actions aimed at preventing tuberculosis among people living with HIV	
*Management is always a problem because we need to convince with each change of government of the importance of the issue.* (P8)		
*We have rapid tests available, but we don’t always reach the most vulnerable populations. And we still have a high rate of treatment abandonment among those diagnosed.* (P17)		
*Centralization is a difficulty and I perceive apprehension from Primary Care in absorbing the care of stable patients.* (P19)		
*I think the partnership we have with the tuberculosis program is very beneficial.* (P20)		
*The control of cases by the specialized service is quite beneficial.* (P23)		

The “Professional qualification and commitment” category highlights the active involvement of professionals, especially nurses, in LTBI screening, diagnosis, and management. There is an appreciation for professional autonomy and acceptance of PTT prescription by non-physicians, expanding access to care. However, challenges arise related to physician resistance to PTT prescription and difficulty in adherence to screening, revealing the importance of strengthening training and ethical commitment in conducting prevention actions (Chart 2).

The “Structure and resources for care” category reveals that the availability of tests, supplies, medications, and technologies is essential for quality care in controlling LTBI. Although there are positive reports on organized systems and access to laboratory tests and therapies, obstacles persist such as a lack of adequate physical spaces, interruptions in material supply, and difficulties in regulating tests. These factors directly impact the continuity and effectiveness of PTT treatment (Chart 3).

The “Treatment adherence and continuity of care” category highlights difficulties in monitoring people living with HIV, especially those experiencing homelessness or with immunosuppression. Low adherence to treatment therapy compromises its effectiveness, even when there is a trained team and resources available. Active outreach, generally limited to telephone contacts, proves ineffective in the face of frequent changes in phone numbers and difficulties in counseling contacts, reinforcing the need for more integrated and sensitive strategies (Chart 4).

The “Health management and policies” category highlights structural and organizational obstacles in the care of LTBI among people living with HIV. The scarcity of qualified professionals, staff turnover, deficient training, and lack of continuous training compromise quality of care. Furthermore, the centralization of care, coupled with political changes and weak coordination between services, hinders continuity of treatment and access to services. Ineffective management limits the effectiveness of public policies (Chart 5).

## DISCUSSION

The findings of this study reveal, on the one hand, the engagement of healthcare teams in the screening and treatment of LTBI, highlighting the role of nursing and the acceptance of clinical practices recommended by national and local guidelines. On the other hand, they point to barriers related to the centralization of the medical figure in prescribing PTT and the resistance of some healthcare professionals to adhere to recommendations due to concerns about polypharmacy, highlighting important obstacles to the effective expansion of access to PTT in Brazil.

A study conducted in 2019 in four Brazilian municipalities and the Federal District analyzed the discourse of 22 healthcare professionals regarding LTBI and observed medical hegemony in the decision regarding (non-)performance of PTT, in contrast to dialogue with the multidisciplinary team and nurses’ and pharmacists’ autonomy^(16)^. Healthcare professionals’ reluctance to prescribe prolonged treatments, with the possibility of adverse effects, to individuals who appear to be healthy, *a priori*, is a problem that needs to be overcome nationally^(17)^.

Although the 3HP regimen, which combines isoniazid and rifapentine administered weekly for three months for PTT, is gaining ground due to its shorter duration and greater practicality, the expansion of this strategy is still far below expectations in the country, since the overlap of treatments (LTBI and HIV) poses a challenge^(16)^. This can be partially attributed to insufficient knowledge of the subject among professionals, which causes fear and insecurity, leading to consequent resistance to recommending treatment^(16)^.

The scientific literature already highlights the importance of strategies that facilitate access to TB prophylaxis, such as proposing new professional categories as prescribers, with a view to increasing the number of people using preventive treatment^(16,18)^. In Brazil, this is already a reality, with physicians, nurses, and pharmacists playing a key role in PTT implementation^(6,18)^. Despite this, this research identified the hegemony of medical professionals’ performance in this area of activity in some scenarios.

Another element that deserves highlighting concerns the insufficient approach to LTBI and PTT in the university training of healthcare professionals. The findings of this study, by highlighting the technical insecurity and resistance of part of the multidisciplinary team in prescribing PTT, indicate a persistent gap in undergraduate and graduate health curricula. Training still privileges a perspective centered on active TB and the biomedical model, with little emphasis on the preventive aspects of TB.

This academic omission can reinforce hierarchical practices, perpetuate the centralization of clinical decisions in the hands of physicians, and limit the role of nurses and pharmacists, even in the face of official regulations that recognize them as qualified prescribers for TB prevention. Therefore, rethinking curricular content in light of current public policies and needs of the Brazilian Unified Health System (In Portuguese, *Sistema Único de Saúde* - SUS) is a fundamental strategy to expand access to TB prevention in priority populations, such as people living with HIV.

The results of this research also highlighted cultural and organizational barriers that reinforce a biomedical and hierarchical model in SCSs, hindering the consolidation of multidisciplinary practices in TB-HIV coinfection care. This configuration not only limits the expansion of access to prophylaxis, but also reproduces a fragmented and disjointed work environment, in which comprehensive care is compromised. This is an even greater challenge in places where specialized care lacks communication with primary health care.

Resistance to the decentralization of prescribing therapy may be rooted in symbolic power struggles, a lack of institutional protocols that legitimize shared practice or the absence of spaces for joint training and integrated decision-making among healthcare professionals. Therefore, in addition to investing in professional development and training, it is necessary to foster a collaborative culture and establish institutional mechanisms that guarantee co-responsibility in therapy implementation in healthcare services.

These findings suggest that the obstacles to PTT implementation are not homogeneous throughout the national territory, since while in some regions structural and logistical barriers predominate, in others, the obstacles seem to be more related to the service and work process organization, the institutional culture, and professional practices. This diversity reinforces the need for regionalized intervention strategies, with specific policies that consider the epidemiological, programmatic, and socioeconomic context.

Similar findings were documented in Ethiopia and South Africa, where shortages of supplies, centralized care, and poor integration between HIV and TB programs also hindered PTT implementation^(18,19)^. Professionals from these countries reported uncertainty regarding clinical indications, fear of polypharmacy, and a lack of ongoing training-common barriers in high-burden TB settings^(18,19)^. This convergence reinforces the idea that the challenge is not structural or global, but rather requires coordination between governments, policies, programs, and teams in each locality.

A study that interviewed over 100 people living with HIV, healthcare professionals, and key informants demonstrated that food insecurity, difficulties taking time off work, and family responsibilities directly impact the decision to initiate PTT^(20)^. The lack of minimum conditions to deal with possible adverse effects, coupled with difficult travel, compromises the acceptance of prophylaxis, even when prescribed. These factors reveal that care is also influenced by social determinants^(20)^.

The findings of this study also demonstrate persistent challenges in the follow-up of people living with HIV in PTT care, especially among those in situations of greater social and economic vulnerability. Participants’ reports reveal that, although resources and qualified teams are available in some locations, effective care is hampered by barriers such as low adherence to treatment, difficulty accessing diagnostic tests, and limitations in communication with people using a therapeutic regimen.

A Brazilian study supported these findings by pointing to the absence of qualified dialogue between professional and user about treatment as a limiting factor in adherence to therapy^(20)^. In Tanzania, low adherence to prenatal care was influenced by HIV-related stigma, posing a challenge to its completion^(19)^. By choosing not to reveal their diagnosis to family and colleagues for fear of judgment or discrimination, their access to support networks, which are fundamental for continuity of care, was compromised^(21)^.

Another relevant aspect for adherence is the incorporation of a shortened therapeutic regimen, which is linked to greater adherence to PTT and a lower number of adverse events, according to research conducted in Campo Grande, Mato Grosso do Sul^(22)^. Regarding the approach to vulnerabilities, the importance of an integrated and coordinated network stands out, with primary health care playing the role of coordinating care^(23)^. Furthermore, considering programmatic factors in infection management requires investment and mobilization by managers^(23,24)^.

For instance, the existence of clusters with a high burden of TB-HIV coinfection and the presentation of increasing trends in disease incidence rates, especially in the North and Northeast regions of the country, points to a possible relationship between worse disease indicators and territorial, social, and programmatic disparities^(6,24,25)^. In this context, policies that increase access to care technologies and establish care pathways should be guaranteed to ensure equitable PTT implementation and maintenance among people living with HIV.

In the context of South Africa, the lack of adequate knowledge and training among professionals regarding the clinical indications for PTT and the lack of integration between TB and HIV services were critical barriers to LTBI screening and treatment^(26)^. In this context, the researchers recommended expanding the training of primary health care professionals and quality improvement strategies, such as organizational changes, multidisciplinary teams, and monitoring and feedback of actions^(26)^.

The importance of horizontal management is reinforced, with policies aimed at solving the structural and organizational problems of services, especially through the coordination of key actors in healthcare services^(27)^. Furthermore, policies that encourage professionals to remain in the SUS and that decentralize and share care across other levels of care are unique strategies that need to be strengthened. Only through collective efforts will it be possible to eliminate TB as a public health problem.

The results presented directly align with Pillar 1 of the End TB Strategy, which emphasizes the essential nature of integrated, person-centered care^(1)^. However, the barriers described-such as irregular access to testing, professional resistance, and poor coordination between primary and specialized care-highlight that there is still a boundary to be crossed between what policy recommends and what is actually implemented in services. Integrating guidelines with practices is crucial for Brazil to move closer to the global goal of offering PTT care to ≥90% of eligible people living with HIV.

Given the complexity of the aspects that limit the prescription of preventive treatment, it is recommended to incorporate mechanisms for continuous monitoring of adherence to national guidelines in SCSs and, eventually, in other healthcare services, with an emphasis on primary health care. Indicators such as the percentage of LTBI screening among people living with HIV, the percentage of prescriptions by professional category, and the percentage of completion of preventive treatment can support more responsive and territorial actions to strengthen preventive treatment within the SUS.

### Study limitations

The limitations of this research include the use of an online approach, which may have hindered the adequate expression of ideas and conceptions of participants, despite the fact that the fields for answering the form were programmed to accept texts of varying lengths, and the possible selection bias, since voluntary participation may have attracted professionals more sensitive to the topic. Furthermore, the absence of field notes which could have contributed to the interpretation of the findings, is considered a major limitation of the research.

### Contributions to nursing, health, or public policy

The findings of this research have direct implications for the practice of managers, HIV and TB program coordinators, and training institutions, since they direct the SUS management spheres, as drivers of the national, state, and municipal response in combating TB, to lead articulated strategies that guarantee territorial equity, continuity of care, and adherence of professionals to current regulations for promoting a TB prevention policy that is integrated and centered on the population’s needs.

## FINAL CONSIDERATIONS

Despite programmatic and technological advancements, significant barriers to PTT implementation for people living with HIV persist in specialized services in Brazil. The findings revealed that resistance to prescription is related not only to resource scarcity and structural limitations, but also to formative, cultural, and organizational obstacles. Technical uncertainty, medical hegemony in decision-making, and low institutionalization of multidisciplinary practice compromise the operationalization of national guidelines.

In this context, structural strategies should be prioritized, including undergraduate health curriculum revision, continuing education program expansion, multidisciplinary practice enhancement, and care decentralization. The adoption of shortened treatment regimens and the coordination between HIV and TB programs are essential to strengthen the healthcare response. Furthermore, public policies must address territorial and programmatic inequalities, with specific investments in the most vulnerable regions.

## Data Availability

The research data are available in a repository: https://doi.org/10.17632/d4cybg9fwh.1.
