# Supplementary material for: Barriers to latent tuberculosis screening and treatment among people living with HIV in Brazil
Source: Rev Bras Enferm. 2026 Aug 21;79:e20250416. doi: 10.1590/0034-7167-2025-0416 (PMC13497369; doi:10.1590/0034-7167-2025-0416)
Supplement: Translation

# Translation: Barreiras ao rastreio e tratamento da tuberculose latente entre pessoas vivendo com HIV no Brasil

**DOI:** 10.1590/0034-7167-2025-0416pt

**Authors:** Eloisa Ganazza Mattera, Lucas Vinícius de Lima, Nelly Lopes de Moraes Gil, Aline Aparecida Monroe, Gabriel Pavinati, Gabriela Tavares Magnabosco

**Affiliations:** I Universidade Estadual de Maringá. Maringá, Paraná, Brasil; II Prefeitura do Município de Maringá, Secretaria Municipal de Saúde. Maringá, Paraná, Brasil; III Universidade de São Paulo. Ribeirão Preto, São Paulo, Brasil

**Keywords:** Tuberculose Latente, HIV, Serviços de Saúde, Terapêutica Preventiva, Pesquisa Qualitativa.

## Abstract

**Objetivos:**

compreender, na perspectiva de profissionais de serviços especializados, os aspectos que influenciam o rastreio e o tratamento da infecção latente pelo *Mycobaterium tuberculosis* entre pessoas vivendo com HIV.

**Métodos:**

estudo qualitativo, com 25 profissionais prescritores do tratamento preventivo de Manaus, Recife, Rio de Janeiro, Campo Grande e Porto Alegre. Os dados foram coletados entre 2024 e 2025, mediante questionário eletrônico, e analisados por meio da análise de conteúdo de Bardin.

**Resultados:**

identificaram-se quatro categorias: Qualificação e compromisso profissional (autonomia e resistência à prescrição); Estrutura e recursos para o cuidado (exames, insumos e infraestrutura); Adesão ao tratamento e continuidade do cuidado (baixa adesão e seguimento clínico); e Gestão e políticas de saúde (escassez de profissionais, rotatividade e centralização).

**Considerações Finais:**

persistem desafios à implementação do tratamento preventivo da tuberculose entre pessoas vivendo com HIV no Brasil, incluindo resistência profissional à prescrição, lacunas formativas, desigualdades regionais no acesso e vulnerabilidades sociais.

## INTRODUÇÃO

A tuberculose (TB) é um dos principais desafios de saúde pública no mundo. Estimativas da Organização Mundial da Saúde indicam que cerca de 25% da população global está infectada pelo bacilo de Koch, mas sem a doença ativa^(1)^. A infecção latente pelo *Mycobacterium tuberculosis* (ILTB), ou TB-infecção, caracteriza-se como uma condição de persistência imunológica do hospedeiro ao bacilo. Historicamente, a ILTB teve menor atenção dos programas de controle da TB, o que dificultou os progressos na redução da cadeia de transmissão^(1-3)^.

Há na literatura o entendimento de que as pessoas com ILTB atuam como “reservatório de bacilos”, com potencial de reativação em situações de imunossupressão^(4,5)^. Esse risco é ainda mais crítico em pessoas vivendo com HIV, devido à imunodeficiência causada pelo vírus, que desfavorece a resposta imunológica do hospedeiro diante do bacilo da TB. Nessas pessoas, a TB não apenas representa a principal causa de morte, mas também um impacto à saúde coletiva, uma vez que aumenta o potencial de disseminação da doença nas comunidades^(5,6)^.

O tratamento preventivo da TB (TPT) assume papel central, pois tem o potencial de reduzir o risco de evolução da ILTB para a forma ativa da TB^(5)^. Revisão de escopo apontou uma redução entre 27% e 95% na incidência de TB-doença entre as pessoas que receberam o TPT em comparação ao grupo placebo, e a eficácia dependeu da duração do tratamento e das taxas de adesão^(7)^. Isso reitera a importância da inclusão do TPT como estratégia crucial no enfrentamento da carga global da TB^(7)^.

A despeito do exposto, obstáculos estruturais e operacionais relacionados aos serviços de saúde, tais como distância até o serviço, ausência de profissionais capacitados, demora no atendimento, imprecisão diagnóstica, baixa adesão ao TPT, cobertura insuficiente do TPT e recursos financeiros insuficientes, são alguns dos desafios apontados na literatura em todo o mundo^(8-11)^ que dificultam o rastreio oportuno da ILTB e, por conseguinte, a implementação do TPT, especialmente em públicos mais vulneráveis, como as pessoas vivendo com HIV.

No Brasil, apesar dos avanços normativos na ampliação do rol de profissionais autorizados a prescrever o TPT - incluindo enfermeiros e farmacêuticos -, persistem resistências à sua implementação. Essa resistência não decorre apenas de barreiras técnicas, mas está relacionada a questões formativas, organizacionais e culturais que limitam a atuação multiprofissional e a efetividade das políticas e estratégias de saúde. Assim, torna-se essencial compreender como as recomendações vigentes se manifestam no cotidiano dos serviços de saúde.

Portanto, compreender os processos de trabalho em diferentes cenários brasileiros permite a identificação dos nós críticos existentes no cotidiano dos serviços, assim como o planejamento de estratégias específicas a cada realidade. Nessa lógica, considerando-se que a qualificação dos profissionais é peça-chave para o alcance das metas globais de eliminação da TB, esta pesquisa se justifica pela necessidade de respaldar ações nacionais voltadas à ampliação da cobertura do TPT entre uma das populações prioritárias nas metas de eliminação da TB.

## OBJETIVOS

Compreender, na perspectiva de profissionais de serviços especializados, os aspectos que influenciam o rastreio e o tratamento da ILTB entre pessoas vivendo com HIV.

## MÉTODOS

### Considerações éticas

A pesquisa prezou pelo atendimento integral às diretrizes da Resolução n.º 466, de 12 de dezembro de 2012, e da Resolução n.º 674, de 6 de maio de 2022, ambas do Conselho Nacional de Saúde. Para tanto, obteve-se a aprovação do Comitê de Ética em Pesquisa com Seres Humanos da Universidade Estadual de Maringá, sob o parecer nº 6.899.096. Também foram obtidas as demais autorizações dos municípios que demandaram a tramitação administrativa e/ou ética. Todos os participantes da pesquisa concordaram com o Termo de Consentimento Livre e Esclarecido para participação no estudo.

### Desenho e contexto do estudo

Pesquisa exploratória, de abordagem qualitativa, relatada em conformidade com o *checklist COnsolidated criteria for REporting Qualitative research* ^(12)^. Para a operacionalização do estudo, foram elencados os Serviços de Atenção Especializada (SAEs) localizados em cinco capitais brasileiras, sendo eles Manaus (Amazonas, na região Norte), Recife (Pernambuco, na região Nordeste), Rio de Janeiro (Rio de Janeiro, na região Sudeste), Campo Grande (Mato Grosso do Sul, na região Centro-Oeste) e Porto Alegre (Rio Grande do Sul, na região Sul).

No Brasil, o tratamento das pessoas vivendo com HIV está organizado de forma compartilhada entre a Atenção Primária à Saúde e a atenção especializada. No entanto, ainda se nota uma centralização nos SAEs, sobretudo nas situações de coinfecção ou múltiplas comorbidades. O SAE é uma unidade ambulatorial que se destina ao manejo do HIV e de outras infecções sexualmente transmissíveis. Mediante atuação multiprofissional, os serviços garantem a prevenção e o tratamento das pessoas acometidas, por meio de cuidado especializado, integral e humanizado.

### Período e participantes do estudo

A coleta de dados ocorreu entre novembro de 2024 e fevereiro de 2025. Foram considerados para o estudo os profissionais de saúde dos SAEs com atuação mínima de seis meses no serviço, que tinham realizado ao menos um atendimento a pessoas vivendo com HIV e possuíam formação compatível com a prescrição do TPT (*i.e*., medicina, enfermagem e farmácia). Destaca-se que esse critério está alinhado à política vigente do Ministério da Saúde, a qual reconhece a possibilidade de médicos, enfermeiros e farmacêuticos orientarem e prescreverem o TPT^(13,14)^.

### Protocolo de coleta de dados

O instrumento utilizado para direcionar a coleta dos dados foi elaborado e adequado por pesquisadores do Grupo de Estudos e Pesquisas em Vigilância do HIV/aids e da Tuberculose da Universidade Estadual de Maringá, responsáveis pelo desenvolvimento do estudo. Foi composto de modo a permitir a caracterização dos participantes, os quais responderam a sete perguntas abertas com o seguinte questionamento central: “Como tem sido o processo de trabalho em relação ao rastreio da ILTB e à indicação do TPT para pessoas vivendo com HIV no seu SAE?”.

Para a captação dos participantes, mais de 250 convites foram realizados, considerando o envio de mensagens diretamente aos contatos institucionais dos profissionais dos SAEs, obtidos pelo *website* do Ministério da Saúde, além de contar com o apoio das coordenações estaduais de programas de TB e HIV e das Secretarias Municipais de Saúde para a divulgação da pesquisa entre os profissionais dos SAEs. O instrumento autoaplicável foi disponibilizado de forma virtual via *Google Forms* ^®^, e, ao final, atingiu-se a saturação teórica com 25 participantes.

Importa pontuar que foram excluídas da pesquisa as respostas ao formulário com textos contendo erros graves de ortografia ou digitação que comprometeram a interpretação parcial ou total do seu conteúdo por parte dos pesquisadores. No total, duas respostas a questões específicas foram desconsideradas por esse motivo. Por fim, esclarece-se que os segmentos textuais foram identificados de acordo com o informante, representado pela letra P (participante), seguido por um número arábico (*e.g*., P1, P2 *etc*.), com vistas a preservar o anonimato.

### Tratamento e análise de dados

O material de texto obtido foi analisado pela técnica da análise de conteúdo de Bardin^(15)^. Essa análise foi ancorada em três etapas sucessivas: pré-análise, em que foram organizados os materiais por meio da leitura flutuante e consequente levantamento de hipóteses; exploração do material, identificando-se as unidades de registro (palavras) e de contexto, por meio de processos de decomposição, classificação, agrupamento e enumeração; e tratamento dos resultados e interpretação, momento em que foi realizada a categorização final.

## RESULTADOS

Dos 25 participantes, a maioria era do sexo feminino (n=23), tinha formação em enfermagem (n=15) e era do Rio de Janeiro (n=12). A idade variou entre 27 e 70 anos (Quadro 1). A partir dos discursos, emergiram nove núcleos de sentido - compostos por elementos constitutivos (discursos dos profissionais) -, que culminaram em quatro categorias: Qualificação e compromisso profissional (Quadro 2); Estrutura e recursos para o cuidado (Quadro 3); Adesão ao tratamento e continuidade do cuidado (Quadro 4); e Gestão e políticas de saúde (Quadro 5).

**Quadro 1:** Descrição das características sociodemográficas e ocupacionais dos participantes da pesquisa, Brasil, 2025

ID	Sexo	Idade (em anos)	Profissão	Mês e ano de início no serviço	Capital
P1	Masculino	49	Enfermeiro	10/2023	Manaus
P2	Masculino	44	Enfermeiro	11/2005	
P3	Feminino	53	Enfermeiro	02/2015	
P4	Feminino	44	Enfermeiro	11/2015	Campo Grande
P5	Feminino	36	Farmacêutico	11/2017	
P6	Feminino	36	Médico	07/2017	Porto Alegre
P7	Feminino	47	Enfermeiro	10/2010	
P8	Feminino	64	Médico	05/2004	
P9	Masculino	38	Farmacêutico	06/2024	Recife
P10	Feminino	27	Enfermeiro	03/2022	
P11	Feminino	37	Enfermeiro	07/2022	
P12	Feminino	27	Enfermeiro	09/2023	
P13	Feminino	42	Enfermeiro	01/2015	
P14	Feminino	64	Médico	11/1991	Rio de Janeiro
P15	Feminino	58	Enfermeiro	10/2022	
P16	Feminino	27	Enfermeiro	01/2024	
P17	Feminino	62	Enfermeiro	09/2022	
P18	Feminino	48	Enfermeiro	08/2013	
P19	Feminino	34	Médico	03/2019	
P20	Feminino	43	Enfermeiro	02/2018	
P21	Feminino	28	Farmacêutico	03/2024	
P22	Feminino	39	Enfermeiro	07/2020	
P23	Feminino	63	Médico	02/2022	
P24	Feminino	40	Médico	02/2023	
P25	Feminino	70	Médico	01/2015	

**Quadro 2:** Apresentação dos núcleos de sentido e elementos constitutivos da categoria “Qualificação e compromisso profissional”, Brasil, 2025

Elementos constitutivos	Núcleos de sentido	Categoria
*Boa parte da equipe está mobilizada a ter atenção e acompanhar os pacientes.* (P9)	O compromisso profissional na prestação da assistência à saúde é uma fortaleza ao diagnóstico e tratamento da tuberculose-infecção	Qualificação e compromisso profissional
*Não tenho muitos problemas na indicação do tratamento preventivo para tuberculose; é muito bem aceito e cumprido pelos profissionais.* (P1)		
*Existe um grande interesse e envolvimento da equipe de enfermagem no controle e manejo da infecção latente.* (P3)		
*A medicação é ofertada a todos os pacientes que atendem às indicações, conforme o protocolo, porque realizamos o rastreio em todos os pacientes junto com a Atenção Primária e com outros serviços de saúde.* (P20)		
*Os enfermeiros realizam o atendimento, a identificação de casos* [...]*, o tratamento, o monitoramento e a busca ativa de pacientes faltosos; isso colabora com o serviço.* (P3)	A autonomia profissional voltada ao rastreio e ao tratamento da tuberculose-infecção outros profissionais amplia o acesso ao cuidado	
*É positiva a autonomia de prescrição do tratamento preventivo por outros profissionais para além dos médicos, especialmente os enfermeiros.* (P7)		
*No nosso serviço, a prescrição do tratamento preventivo é uma conduta médica, e não são todos os colegas que seguem as recomendações por justificativas associadas à polifarmácia.* (P9)	A resistência profissional à prescrição do tratamento preventivo da tuberculose dificulta o cumprimento das recomendações vigentes	
*A conduta médica em relação à prescrição do tratamento preventivo da tuberculose é um desafio.* (P12)		
*O rastreio não é feito como deveria* [...]; *observo uma “dificuldade” dos profissionais médicos em solicitar o rastreio da tuberculose latente.* (P18)		

**Quadro 3:** Apresentação dos núcleos de sentido e elementos constitutivos da categoria “Estrutura e recursos para o cuidado”, Brasil, 2025

Elementos constitutivos	Núcleos de sentido	Categoria
*Temos meios de monitoramento sistematizados em planilhas que auxiliam na busca ativa daqueles que interrompem o tratamento.* (P10)	A (in)disponibilidade de recursos físicos e de tecnologias de cuidado é parte indispensável para uma assistência de qualidade	Estrutura e recursos para o cuidado
*O ponto forte é o acompanhamento do CD4 como indicativo para início do tratamento.* (P1)		
*A incorporação de testes diagnósticos* [da infecção latente da tuberculose] *para pessoas vivendo com HIV amplia o acesso.* (P5)		
*Temos no nosso serviço a constante disponibilidade dos medicamentos necessários* [...]*, o que não é uma realidade em todos os lugares.* (P6)		
*Tenho acesso rápido a exames de imagem, como radiografia e tomografia computadorizada, e exames laboratoriais, como teste rápido molecular para tuberculose, além de equipe de saúde treinada.* (P8)		
*Ter acesso ao LF-LAM* [teste de fluxo lateral para detecção de lipoarabinomanano em urina] *é uma grande vantagem na rotina, porém ainda temos dificuldade em relação à radiografia, pois precisamos ficar encaminhando para a regulação.* (P12)	A (in)disponibilidade de recursos físicos e de tecnologias de cuidado é parte indispensável para uma assistência de qualidade	Estrutura e recursos para o cuidado
*Fomos desvinculados da nossa unidade ficando em uma casa sem condições do usuário ser atendido na sua plenitude* [...]. *Nossa médica ficou sem contrato e, no momento, estamos aguardando a recontratação para continuidade dos tratamentos.* (P15)		
*Nós temos laboratório na epidemiologia* [...], *temos vários profissionais multiplicadores da prova tuberculínica. Resultados de exames são quase imediatos. A equipe multiprofissional integra o serviço especializado e faz as ações necessárias.* (P14)		
*A quantidade de comprimidos* [...], *ainda não temos o esquema 3HP disponível para todos os pacientes com a indicação.* (P7)	Os problemas referentes à disponibilidade de insumos e estrutura física dificultam o tratamento da tuberculose-infecção	
*Infelizmente, temos um espaço físico inadequado às necessidades.* (P14)		
*Às vezes, não temos disponibilidade de exames como prova tuberculínica e IGRA* [*interferon gamma release assay*] *no momento zero do atendimento.* (P22)		
*Nosso espaço físico é restrito; isso limita a assistência.* (P20)		

**Quadro 4:** Apresentação dos núcleos de sentido e elementos constitutivos da categoria “Adesão ao tratamento e continuidade do cuidado”, Brasil, 2025

Elementos constitutivos	Núcleos de sentido	Categoria
*O desafio no rastreio é garantir que os pacientes façam a radiografia de tórax que foi solicitada.* (P2)	A baixa adesão das pessoas vivendo com HIV à terapia prejudica a efetividade do tratamento preventivo, sobrepondo-se às vulnerabilidades sociais	Adesão ao tratamento e continuidade do cuidado
*Temos bastante dificuldades principalmente com aqueles pacientes que estão em situação de rua.* (P3)		
*Adesão do paciente ao tratamento: isso é um problema que temos muito por aqui.* (P8)		
[...] *existem casos de pacientes de adesão difícil.* (P10)		
*Apesar da equipe qualificada e disponibilidade de insumo, temos vários pacientes que não aderem ao tratamento preventivo.* (P25)		
*O principal desafio* [...] *é a observação do paciente após início da terapia, pensando especialmente naqueles pacientes* [...] *com CD4 menor que 350 células/milímetros cúbicos.* (P6)	Os desafios relacionados à busca ativa e ao aconselhamento dos indivíduos em uso do tratamento preventivo e seus contatos	
*Costumamos realizar a busca ativa somente via celular, e isso não é muito efetivo* [...]; *os números de telefones mudam muito.* (P1)		
*Acessar e aconselhar os contatos é desafiador, uma dificuldade.* (P24)		

**Quadro 5:** Apresentação dos núcleos de sentido e elementos constitutivos da categoria “Gestão e políticas de saúde”, Brasil, 2025

Elementos constitutivos	Núcleos de sentido	Categoria
*Ausência de recursos humanos e profissionais qualificados.* (P3)	Os profissionais em quantidade insuficiente, com formação deficitária e pouco qualificados obstaculizam a implementação do tratamento preventivo	Gestão e políticas de saúde
*O tempo de espera para uma consulta pode ser demorado.* (P9)		
*O rodízio de profissionais contratados e as trocas periódicas dos profissionais da rede.* (P8)		
*Os profissionais recém-formados têm pouco conhecimento sobre o tema; isso prejudica a assistência.* (P8)		
*Para mim, vejo que falta de conhecimento na hora de decidir sobre a indicação do tratamento preventivo.* (P11)		
*Temos uma equipe pequena para a demanda.* (P14)		
*Precisamos melhorar muito ainda na capacitação dos profissionais.* (P16)		
*Bastante frágil o fato de a disponibilização do cuidado à tuberculose-infecção ser centralizado em pontos da rede.* (P7)	Os desafios políticos e organizacionais dificultam a oferta de serviços e ações destinadas à prevenção da tuberculose entre pessoas vivendo com HIV	
*A gestão é sempre um problema porque precisamos convencer a cada mudança de governo a importância do tema.* (P8)		
*Temos testes rápidos disponíveis, mas nem sempre alcançamos as populações mais vulneráveis. E ainda temos tido alta taxa de abandono dos tratamentos entre os diagnosticados.* (P17)		
*A centralização é uma dificuldade, e percebo receio da atenção básica em absorver o atendimento dos pacientes estáveis.* (P19)		
*Acho que a parceria que temos com o programa de tuberculose é muito benéfica*. (P20)		
*O controle dos casos pelo serviço especializado é bastante benéfico.* (P23)		

A categoria “Qualificação e compromisso profissional” evidencia o envolvimento ativo de profissionais, especialmente da enfermagem, no rastreio, diagnóstico e manejo da ILTB. Observam-se a valorização da autonomia profissional e a aceitação da prescrição do TPT por não médicos, ampliando o acesso ao cuidado. Entretanto, surgem desafios relacionados à resistência médica à prescrição do TPT e à dificuldade de adesão ao rastreamento, revelando a importância de fortalecer a formação e o compromisso ético na condução das ações de prevenção (Quadro 2).

A categoria “Estrutura e recursos para o cuidado” revela que a disponibilidade de exames, insumos, medicamentos e tecnologias é essencial para uma assistência de qualidade no controle da ILTB. Embora haja relatos positivos sobre sistemas organizados e acesso a testes laboratoriais e terapias, persistem obstáculos como carência de espaços físicos adequados, interrupções no fornecimento de insumos e entraves na regulação de exames. Esses fatores impactam diretamente a continuidade e a efetividade do TPT (Quadro 3).

A categoria “Adesão ao tratamento e continuidade do cuidado” evidencia dificuldades no seguimento de pessoas vivendo com HIV, especialmente aquelas em situação de rua ou com imunossupressão. A baixa adesão ao TPT compromete sua efetividade, mesmo quando há equipe capacitada e insumos disponíveis. A busca ativa, geralmente limitada a contatos telefônicos, mostra-se ineficaz diante de mudanças frequentes de número e dificuldades no aconselhamento de contatos, reforçando a necessidade de estratégias mais integradas e sensíveis (Quadro 4).

A categoria “Gestão e políticas de saúde” evidencia entraves estruturais e organizacionais no cuidado à ILTB entre pessoas vivendo com HIV. A escassez de profissionais qualificados, rotatividade nas equipes, formação deficitária e ausência de capacitação contínua comprometem a qualidade da assistência. Além disso, a centralização do cuidado, aliada a mudanças políticas e à fragilidade da articulação entre serviços, dificulta a continuidade do tratamento e o acesso aos serviços. A gestão ineficaz limita a efetividade das políticas públicas (Quadro 5).

## DISCUSSÃO

Os achados do presente estudo revelam, de um lado, o engajamento das equipes de saúde no rastreio e no tratamento da ILTB, com destaque para a atuação da enfermagem e a aceitação das condutas clínicas recomendadas por diretrizes nacionais e locais. Por outro lado, apontam para barreiras relacionadas à centralização da figura médica na prescrição do TPT e à resistência de parte dos profissionais de saúde em aderir às recomendações por receio quanto à polifarmácia, evidenciando obstáculos importantes para a efetiva ampliação do acesso ao TPT no Brasil.

Pesquisa conduzida em 2019 em quatro municípios brasileiros e no Distrito Federal analisou o discurso de 22 profissionais de saúde sobre a ILTB e observou a hegemonia médica na decisão quanto à (não) realização do TPT, em oposição ao diálogo com a equipe multiprofissional e à autonomia de enfermeiros e farmacêuticos^(16)^. A relutância de profissionais de saúde em prescrever tratamentos prolongados, com possibilidade de efeitos adversos, em indivíduos que aparentam estar saudáveis, *a priori,* é um problema a ser superado nacionalmente^(17)^.

Muito embora o regime 3HP, que combina isoniazida e rifapentina administrados semanalmente por três meses para o TPT, esteja ganhando espaço devido à menor duração e à maior praticidade, a expansão dessa estratégia ainda está muito aquém do esperado no país, uma vez que a sobreposição de tratamento (ILTB e HIV) se impõe como uma desafio^(16)^. Parcialmente, isso pode ser atribuído ao conhecimento insuficiente dos profissionais sobre a temática, o que provoca medo e insegurança, gerando a consequente resistência à indicação do tratamento^(16)^.

Já está destacada na literatura científica a importância de estratégias que facilitem o acesso à profilaxia da TB, a exemplo da proposição de novas categorias profissionais como prescritoras, com vistas a aumentar o número de pessoas em uso do TPT^(16,18)^. No Brasil, isso já é uma realidade, com médicos, enfermeiros e farmacêuticos sendo atores elementares na implementação do TPT^(6,18)^. Apesar disso, esta pesquisa identificou a hegemonia da atuação dos profissionais de medicina nessa frente de atuação em alguns cenários.

Outro elemento que merece destaque diz respeito à insuficiente abordagem da ILTB e do TPT na formação universitária dos profissionais de saúde. Os achados deste estudo, ao evidenciarem a insegurança técnica e a resistência de parte da equipe multiprofissional na prescrição do TPT, indicam uma lacuna persistente nos currículos de graduação e pós-graduação em saúde. A formação ainda privilegia uma perspectiva centrada na TB ativa e no modelo biomédico, com pouca ênfase nos aspectos preventivos da TB.

Essa omissão acadêmica pode reforçar práticas hierarquizadas, perpetuar a centralização das decisões clínicas na figura médica e limitar a atuação de enfermeiros e farmacêuticos, mesmo diante de normativas oficiais que os reconhecem como prescritores aptos ao TPT. Portanto, repensar os conteúdos curriculares à luz das políticas públicas vigentes e das necessidades do Sistema Único de Saúde (SUS) é uma estratégia fundamental para ampliar o acesso à prevenção da TB em populações prioritárias, como as pessoas vivendo com HIV.

Os resultados desta pesquisa também evidenciaram barreiras culturais e organizacionais que reforçam um modelo biomédico e hierarquizado nos SAEs, dificultando a consolidação de práticas multiprofissionais no cuidado à coinfecção TB-HIV. Essa configuração não apenas limita a ampliação do acesso à profilaxia, mas também reproduz um ambiente de trabalho fragmentado e desarticulado, em que o cuidado integral é comprometido. Isso é um desafio ainda maior em locais onde a atenção especializada não dispõe de comunicação com a atenção primária à saúde.

A resistência à descentralização da prescrição do TPT pode estar ancorada em disputas simbólicas de poder, na falta de protocolos institucionais que legitimem a atuação compartilhada ou na ausência de espaços de formação conjunta e tomada de decisão integrada entre os profissionais de saúde. Assim, além do investimento em capacitação e formação dos profissionais, é necessário fomentar a cultura colaborativa e instituir mecanismos institucionais que garantam a corresponsabilidade na implementação do TPT nos serviços de saúde.

Esses achados sugerem que os entraves à implementação do TPT não são homogêneos em todo o território nacional, pois enquanto em algumas regiões predominam barreiras estruturais e logísticas, em outras, os obstáculos parecem estar mais relacionados à organização do serviço e do processo de trabalho, à cultura institucional e a práticas profissionais. Tal diversidade reforça a necessidade de estratégias regionalizadas de intervenção, com políticas específicas que considerem o contexto epidemiológico, programático e socioeconômico.

Achados similares foram documentados na Etiópia e na África do Sul, onde a escassez de insumos, a centralização do cuidado e a pouca integração entre programas de HIV e TB também dificultaram a implementação do TPT^(18,19)^. Profissionais desses países relataram insegurança quanto às indicações clínicas, receio da polifarmácia e ausência de treinamento contínuo - barreiras comuns em contextos de alta carga de TB^(18,19)^. Essa convergência reforça que o desafio é estrutural e global, o que demanda coordenação entre governos, políticas, programas e equipes de cada localidade.

Estudo que entrevistou mais de 100 pessoas vivendo com HIV, profissionais de saúde e informantes-chave demonstrou que insegurança alimentar, dificuldades para se ausentar do trabalho e responsabilidades familiares impactam diretamente a decisão em iniciar o TPT^(20)^. A ausência de condições mínimas para lidar com possíveis efeitos adversos, somada ao deslocamento dificultado, compromete a aceitação da profilaxia, mesmo quando prescrita. Esses fatores revelam que o cuidado é atravessado também pela determinação social^(20)^.

Os achados do presente estudo demonstram, ainda, desafios persistentes no seguimento das pessoas vivendo com HIV em TPT, especialmente entre aquelas em situação de maior vulnerabilidade social e econômica. Os relatos dos participantes revelam que, embora haja disponibilidade de insumos e equipes qualificadas em alguns locais, a efetivação do cuidado é dificultada por barreiras como baixa adesão ao tratamento, dificuldade no acesso a exames diagnósticos e limitações na comunicação com as pessoas em uso do esquema terapêutico.

Estudo brasileiro ratificou tais achados ao apontar a ausência de diálogo qualificado entre profissional-usuário sobre o tratamento como um limitador da adesão ao TPT^(20)^. Na Tanzânia, a baixa adesão ao TPT foi influenciada pelo estigma relacionado ao HIV, configurando-se como um desafio para sua conclusão^(19)^. Ao optarem por não revelar seu diagnóstico a familiares e colegas por receio de julgamento ou discriminação, o acesso a redes de apoio, as quais se mostram como fundamentais para continuidade do cuidado, foi prejudicado^(21)^.

Outro aspecto relevante para a adesão é a incorporação de um regime terapêutico encurtado que está atrelado a uma maior adesão ao TPT e a um menor número de eventos adversos, conforme pesquisa realizada em Campo Grande, Mato Grosso do Sul^(22)^. No que se refere à abordagem das vulnerabilidades, destaca-se a importância de uma rede integrada e articulada, com a atenção primária à saúde desempenhando o papel de ordenadora do cuidado^(23)^. Além disso, considerar fatores programáticos no manejo da infecção exige investimentos e mobilização pelos gestores^(23,24)^.

Por exemplo, a existência de *clusters* de alta carga da coinfecção TB-HIV e a apresentação de tendências crescentes dos coeficientes de incidência da doença, sobretudo nas regiões Norte e Nordeste do país, direcionam a possível relação entre piores indicadores da doença e disparidades territoriais, sociais e programáticas^(6,24,25)^. Nessa vertente, políticas que aumentem o acesso às tecnologias de cuidado e estabeleçam fluxos assistenciais devem ser garantidas em prol da implementação e manutenção igualitária do TPT entre as pessoas com HIV.

No contexto da África do Sul, a falta de conhecimento e treinamento adequados pelos profissionais em relação às indicações clínicas do TPT, bem como a não integração entre serviços de TB e HIV, foram barreiras críticas para o rastreio e o tratamento da ILTB^(26)^. Nesse contexto, os pesquisadores recomendaram a expansão da capacitação de profissionais de atenção primária à saúde e estratégias de melhoria da qualidade, tais como mudanças organizacionais, equipes multidisciplinares, monitoramento e *feedback* das ações^(26)^.

Reforça-se a importância de uma gestão horizontal, com políticas que visam solucionar os problemas estruturais e organizacionais dos serviços, especialmente com a articulação dos atores-chave dos serviços de saúde^(27)^. Ainda, políticas de incentivo à permanência de profissionais ao SUS e de descentralização e compartilhamento do cuidado em outros níveis de atenção são estratégias ímpares e que precisam ser fortalecidas. Somente mediante esforços coletivos será possível alcançar a eliminação da TB como problema de saúde pública.

Os resultados evidenciados dialogam de maneira direta com o pilar 1 da *End TB Strategy*, que enfatiza a essencialidade de cuidados integrados e centrados na pessoa^(1)^. No entanto, as barreiras descritas - como acesso irregular a exames, resistência profissional e baixa articulação entre atenção primária à saúde e especializada - evidenciam que ainda há uma fronteira a ser transposta entre o que a política recomenda e o que se operacionaliza nos serviços. Integrar as diretrizes às práticas é crucial para que o Brasil se aproxime da meta global de ofertar TPT para ≥90% das pessoas vivendo com HIV elegíveis.

Diante da complexidade dos aspectos que limitam a prescrição do TPT, recomenda-se a incorporação de mecanismos de monitoramento contínuo da adesão às diretrizes nacionais nos SAEs e, eventualmente, nos demais serviços de saúde, com ênfase na atenção primária à saúde. Indicadores como percentual de rastreio de ILTB entre as pessoas vivendo com HIV, percentual de prescrição por categoria profissional e percentual de conclusão do tratamento preventivo podem subsidiar ações mais responsivas e territoriais para o fortalecimento do TPT no SUS.

### Limitações do estudo

As limitações desta pesquisa incluíram o uso da coleta *online*, que pode ter prejudicado a adequada exposição de ideias e concepções dos participantes, apesar de os campos para respostas ao formulário terem sido programados para aceitarem textos de tamanhos variados, e o possível viés de seleção, visto que a participação voluntária pode ter atraído profissionais mais sensibilizados com o tema. Ainda, entende-se como maior restrição da pesquisa a ausência de registros de campo, que poderiam ter contribuído para a interpretação dos achados.

### Contribuições para a área da enfermagem, saúde ou políticas públicas

Os achados desta pesquisa têm implicações diretas para a prática de gestores do SUS, coordenadores de programas de HIV e TB, e instituições formadoras, uma vez que direcionam às esferas de gestão do SUS, enquanto indutoras da resposta nacional, estadual e municipal no enfrentamento da TB, a liderar estratégias articuladas que garantam equidade territorial, continuidade do cuidado e adesão dos profissionais às normativas vigentes para a promoção de uma política de prevenção da TB que seja integrada e centrada nas necessidades da população.

## CONSIDERAÇÕES FINAIS

Apesar dos avanços programáticos e tecnológicos, persistem importantes barreiras à implementação do TPT para pessoas vivendo com HIV nos serviços especializados do Brasil. Os achados revelaram que a resistência à prescrição está relacionada não apenas à escassez de recursos e a limitações estruturais, mas também a entraves formativos, culturais e organizacionais. A insegurança técnica, a hegemonia médica nas decisões e a baixa institucionalização da prática multiprofissional comprometem a operacionalização das diretrizes nacionais.

Nesse contexto, estratégias estruturantes devem ser priorizadas, incluindo a revisão dos currículos da graduação em saúde, a ampliação de programas de educação permanente, a valorização da atuação multiprofissional e a descentralização do cuidado. A adoção de regimes terapêuticos encurtados e a articulação entre programas de HIV e TB são essenciais para fortalecer a resposta assistencial. Além disso, políticas públicas devem se atentar às desigualdades territoriais e programáticas, com investimento específico nas regiões de maior vulnerabilidade.

## Data Availability

Os dados de pesquisa estão disponíveis em repositório: https://doi.org/10.17632/d4cybg9fwh.1.
